# Systematic review and meta-analysis of the effectiveness of rehabilitation interventions on upper limb function in patients with systemic sclerosis

**DOI:** 10.3389/fmed.2026.1899005

**Published:** 2026-09-09

**Authors:** Qiao Hu, Ting Yao, Miaomiao Li, Xiang Wang

**Affiliations:** 1 The School of Nursing, Anhui University of Chinese Medicine, Hefei, China; 2 Laboratory of Geriatric Nursing and Health, Anhui University of Chinese Medicine, Hefei, China

**Keywords:** meta-analysis, rehabilitation exercises, systematic review, systemic sclerosis, upper limb

## Abstract

**Objective:**

To systematically synthesize the available evidence regarding the effects of rehabilitation interventions on upper limb function, pain and activity performance in patients with systemic sclerosis (SSc), and to further evaluate the limitations of current evidence base and the comparative effectiveness of rehabilitation delivery modes.

**Methods:**

Computer searches of databases such as PubMed, Embase, Web of Science, The Cochrane Library, Medline, CINAHL, the SinoMed (China Biomedical Literature Service System), CNKI, WanFang Data Knowledge Service Platform,and VIP were conducted to collect randomized controlled trials on rehabilitation interventions for upper limb function in patients with systemic sclerosis, with search time spanning from database inception (January 1, 1990) to May 2026. Considering the limited number, high clinical heterogeneity and generally moderate-to-high methodological bias risk of included trials, this study adopted a combination of meta-analysis and cautious narrative synthesis. Two researchers independently screened the literature and extracted data according to inclusion and exclusion criteria, and assessed the quality of included studies using the bias risk assessment tool recommended in the Cochrane Handbook 5.1.0. Meta-analysis was performed using RevMan 5.4 software for outcomes with acceptable heterogeneity.

**Results:**

A total of 8 studies were included, with a total of 729 cases. The overall evidence base is limited, with no high-quality low-bias Grade A studies, and a single large trial dominates the pooled weight of many analyses. Meta-analysis results showed that patients with systemic sclerosis who received rehabilitation intervention demonstrated significant improvements in pain [MD = −4.41, 95% CI (−6.54, −2.29), *P* < 0.0001], hand mobility [MD = −2.58, 95% CI (−4.09, −1.06), *P* = 0.0009], and DASH score [MD = −6.91, 95% CI (−12.54, −1.27), *P* = 0.02] at the end of intervention compared to the control group, with statistically significant differences. For outcome indicators that could be subjected to subgroup analysis, the results showed that face-to-face rehabilitation guidance yielded a marginally significant improvement in hand function [SMD = −0.40, 95% CI (−0.77, −0.02), *P* = 0.04]; however, such subgroup findings should be interpreted cautiously due to small sample size and confounding factors. Meanwhile, the effect of rehabilitation exercises on improving patients' SHAQ scores was not significant [MD = −0.03, 95% CI (−0.25, 0.20), *P* = 0.81], with no statistically significant difference.

**Conclusion:**

Rehabilitation interventions have a positive effect on the hand function, hand mobility, and local upper limb function of patients with systemic sclerosis, and can also aid in pain relief. However, rehabilitation cannot improve the multidimensional overall disease status reflected by SHAQ. Current evidence is limited by small trial quantity, high heterogeneity and suboptimal methodological quality; firm definitive conclusions and practice-changing recommendations cannot be drawn. Face-to-face rehabilitation displays only preliminary favorable signals compared with remote rehabilitation, and definite superiority cannot be verified. In clinical practice, rehabilitation programs may be selected based on individual patient circumstances with very cautious reference to the limited evidence base.

**Systematic Review Registration:**

https://www.crd.york.ac.uk/prospero/display_record.php?RecordID=1019908, PROSPERO CRD420251019908.

## 1 Introduction

Systemic sclerosis (SSc), also known as scleroderma, is an immune-mediated rheumatic disease characterized primarily by fibrosis of the skin and internal organs, as well as vasculopathy (1, 2). SSc can be classified into two subtypes: limited cutaneous systemic sclerosis and diffuse cutaneous systemic sclerosis (3). According to statistics, approximately 90% of SSc patients experience impaired hand function, manifesting as hand contractures, reduced range of motion of joints, tendinopathy, and muscle weakness. In addition, patients often suffer from pain and cutaneous manifestations. These symptoms not only significantly compromise patients' daily living and occupational functioning but may also lead to psychological disturbances such as depression, frustration, and anxiety, resulting in reduced quality of life (4–6). Beyond local upper limb impairment, SSc involves multisystem lesions including gastrointestinal, pulmonary and peripheral vascular involvement, which collectively affect patients' overall health and long-term prognosis.

Current management of SSc relies heavily on pharmacotherapy to target specific symptoms and organ involvement. However, drug therapy has notable limitations, including a high incidence of adverse effects, considerable variability in individual treatment response, and high treatment costs (7). Consequently, there is a growing interest in non-pharmacological approaches, such as rehabilitation interventions, to address functional limitations. Rehabilitation offers a promising complementary strategy, with potential to alleviate symptoms, restore function, and improve overall well-being, thereby mitigating some limitations of purely pharmacological management (8–11).

Although several clinical trials have investigated the role of rehabilitation in improving upper limb function in SSc, their findings are inconsistent, and their individual sample sizes are often insufficient to draw definitive conclusions. Importantly, the existing RCTs are highly heterogeneous in intervention type, training intensity, intervention duration and control setting; moreover, most trials are of suboptimal methodological quality, lacking low risk-of-bias design. A single large trial often dominates the quantitative pooling, making traditional meta-analysis prone to overinterpretation. This heterogeneity, limited sample volume and methodological flaws preclude a clear consensus on the efficacy of these interventions. Only a few prior systematic reviews covered rehabilitation for SSc, yet none stratified analyses to compare face-to-face vs. remote rehabilitation. The present meta-analysis addresses this gap. Face-to-face and remote rehabilitation differ greatly in supervision, operability and patient compliance; clarifying their respective effects can guide clinicians to select suitable rehabilitation modes for different patients. Therefore, we conducted this systematic review and meta-analysis supplemented with cautious narrative synthesis to synthesize the existing evidence, objectively appraise evidence limitations, and provide a robust but prudent quantitative evaluation of the effectiveness and safety of rehabilitation interventions for upper limb function in patients with SSc.

## 2 Materials and methods

This systematic review and meta-analysis was conducted in accordance with the Cochrane Handbook for Systematic Reviews of Interventions and reported following the PRISMA (Preferred Reporting Items for Systematic Reviews and Meta-Analyses) statement. The study protocol was registered on PROSPERO (CRD 420251019908). Given the inherent limitations of the included evidence, we adopted restrained statistical pooling and avoided over-interpreting quantitative results.

### 2.1 Literature inclusion and exclusion criteria

The eligibility criteria were defined based on the PICOS framework:

Population: Patients aged ≥18 years who met the 2013 American College of Rheumatology/European League Against Rheumatism classification criteria for systemic sclerosis (12).

Intervention: The experimental group received structured upper-limb rehabilitation interventions, which were pre-classified into two distinct biomechanical categories for stratified meta-analysis: ① passive mechanical interventions, including connective tissue massage, McMennell joint manipulation and manual joint mobilization; ② active motor training, including supervised or remote home stretching, resistance and hand functional exercise delivered via internet or telephone guidance. Due to fundamental differences in tissue loading mechanisms between passive and active rehabilitation modalities, all quantitative syntheses were planned to be stratified by intervention type *a priori*. Subgroup analyses were conducted where adequate studies were available to avoid pooling clinically heterogeneous treatment approaches into a single generic effect estimate.

Comparison: The control group received conventional home-based exercise or usual care. Notably, the definition of “conventional” control varied considerably across individual studies without standardized criteria, which may reduce the accuracy of effect size estimation. During data extraction, we recorded the specific operational definition of conventional home-based exercise or usual care in each trial. If heterogeneous definitions led to unacceptable clinical heterogeneity, meta-analytic pooling would be avoided. We stratified included trials based on the consistency of hand exercise regimens between groups to separate confounding effects derived from training content and supervision, and only pooled studies with fully matched exercise schemes to assess supervision efficacy.

Outcome: At least one of the following outcome measures was included. We predefined a clinical hierarchy of outcomes:the primary outcome was the Cochin Hand Function Scale (CHFS) focusing on local hand function. Secondary outcomes included the Visual Analog Scale (VAS) for pain, the Hand Mobility in Scleroderma (HAMIS) test, and the Disabilities of the Arm, Shoulder and Hand (DASH) questionnaire for upper limb-specific disability. The Scleroderma Health Assessment Questionnaire (SHAQ) was regarded only as a supplementary exploratory global outcome, as it reflects multisystem disease burden rather than isolated upper limb function. We included this scale to capture patients' overall daily disability instead of evaluating visceral organ recovery.

Study design: Randomized controlled trials (RCTs).

Studies were excluded if they were conference abstracts, animal studies, or if the full text was unavailable and sufficient data could not be obtained from the authors.

### 2.2 Search strategy

A systematic literature search was performed from inception until May 2026 in the following electronic databases: PubMed, Embase, Web of Science, The Cochrane Library (CENTRAL), CINAHL, SinoMed, CNKI, WanFang Database, and VIP. The search strategy utilized a combination of Medical Subject Headings (MeSH) and free-text terms related to “systemic sclerosis”, “scleroderma”, “rehabilitation”, “exercise therapy”, and “upper extremity”. The search strategy for PubMed is provided in Supplementary Figure 1. No language restrictions were initially applied, and ultimately only English and Chinese literatures were included, while studies published in other languages were excluded.

### 2.3 Study selection and data extraction

Two investigators who had received training in evidence-based medicine methods independently performed literature screening and data extraction according to predefined inclusion and exclusion criteria, followed by cross-verification. Any disagreements were resolved through discussion; if consensus could not be reached, a third investigator was consulted for arbitration. A uniformly designed data extraction form was used to collect the following information: basic information, including the title of the article, first author, year of publication, and country; baseline characteristics of the study subjects, such as sample size and disease type; interventions or exposure factors in the experimental and control groups; and outcome measures. We noted that most subgroup and outcome analyses relied on only 2–3 studies, with one large trial carrying excessive weight in pooled estimates.

### 2.4 Methodological quality assessment

Two investigators independently assessed the quality of the included studies using the risk of bias (ROB) evaluation tool recommended by the Cochrane Handbook 5.1.0 (13). The assessment covered seven domains: random sequence generation, allocation concealment, blinding of participants and personnel, blinding of outcome assessors, completeness of outcome data (attrition), selective reporting, and other potential sources of bias. Each item was rated as “low risk,” “high risk,” or “unclear risk” of bias. Any disagreements were resolved through discussion, and if consensus could not be reached, a third investigator made the final decision. For studies with incomplete or missing outcome data, we attempted to contact the corresponding authors to obtain raw original data for quantitative pooling before final exclusion. Based on the fulfillment of the criteria, the studies were classified into three categories: those meeting all criteria were classified as Grade A, indicating low risk of bias and high quality; those partially meeting the criteria were classified as Grade B, indicating moderate risk of bias; and those meeting none of the criteria were classified as Grade C, indicating high risk of bias. Studies categorized as Grade C were excluded from this analysis. Notably, none of the included studies achieved Grade A high-quality low-bias level; all were Grade B, with nearly half presenting high or unclear risk in key domains, which substantially limits the credibility of pooled results.

### 2.5 Statistical analysis

Meta-analysis of the included studies was performed using RevMan software (version 5.4). For continuous outcomes, the mean difference (MD) or standardized mean difference (SMD) was used; for dichotomous outcomes, the relative risk (*RR*) was applied, along with their corresponding 95% confidence intervals (CIs). Heterogeneity among studies was assessed using the *I*^2^ statistic. An *I*^2^ value of less than 50% was considered to indicate acceptable heterogeneity, and a fixed-effects model based on the Mantel-Haenszel method was employed for the meta-analysis. If *I*^2^ was 50% or greater, significant heterogeneity was considered present, and a random-effects model based on the DerSimonian and Laird method was used. In cases of substantial heterogeneity, a sensitivity analysis was conducted by sequentially removing individual studies to explore potential sources of heterogeneity. Given that the included study number was far less than 10, funnel plot results were interpreted cautiously rather than overstated for publication bias inference.

## 3 Results

### 3.1 Literature screening results

A total of 1,140 records were identified through the initial search. After primary and secondary screening, 8 studies were ultimately included. The literature selection process is illustrated in Supplementary Figure 2.

### 3.2 Basic characteristics and quality assessment of included studies

A total of 8 studies (14–21) were included, involving 424 patients in the experimental group and 305 in the control group, with a total sample size of 729 participants. All included studies reported clear outcome measures and provided detailed descriptions of the interventions. The basic characteristics are summarized in Supplementary Table. The quality of all included studies was rated as Grade B, with no Grade A high-quality trials available. Seven studies (14–16, 18–21) provided a detailed description of the randomization method. Three studies (15, 18, 19) described the method of allocation concealment in detail. Three studies (15, 18, 20) reported cases of loss to follow-up, while the remaining studies had complete outcome data. No selective outcome reporting bias was detected in any of the studies. Three studies (15, 17, 18) were identified with other potential sources of bias. See Supplementary Figures 3, 4 for details. It should be emphasized that many comparative analyses were based on merely two studies and were heavily influenced by a single dominant trial, limiting generalizability.

### 3.3 Meta-analysis results

Given the limited number of included trials, small subgroup sample sizes and moderate overall methodological quality, all pooled effect estimates reported in this meta-analysis should be interpreted with extreme caution.

#### 3.3.1 Effect of rehabilitation interventions on hand function in patients with SSc

Five studies (14, 15, 17–19) reported the effects of rehabilitation interventions on hand function. Based on the mode of exercise implementation, the studies were divided into two subgroups: a telerehabilitation exercise group (15, 18) and an in-person face-to-face rehabilitation guidance group (14, 17, 19). The results showed that the pooled effect size for telerehabilitation exercise on hand function in patients with SSc was [SMD = −0.04, 95% CI (−0.22, 0.14), *P* = 0.65], indicating that telerehabilitation exercise did not significantly improve hand function. In contrast, the pooled effect size for in-person face-to-face rehabilitation guidance on hand function was [SMD = −0.40, 95% CI (−0.77, −0.02), *P* = 0.04], suggesting a marginally favorable trend of in-person rehabilitation. Nevertheless, due to small subgroup sample size, clinical heterogeneity in supervision intensity and patient adherence, this finding cannot confirm definite superiority of face-to-face over remote rehabilitation and should only be regarded as a tentative observation, as shown in Supplementary Figure 5. Notably, our predefined subgroup division (in-person rehabilitation vs. remote telerehabilitation) inherently separates passive manual mechanical therapy and unsupervised active home training, thus avoiding inappropriate pooling of biomechanically divergent interventions into one overall effect size.

Notably, the favorable pooled trend observed for in-person rehabilitation cannot be attributed solely to on-site supervision, as it is confounded by additional manual physiotherapy provided only to intervention groups.

#### 3.3.2 Effect of rehabilitation intervention on hand pain in patients with SSc

**Figure 1:**
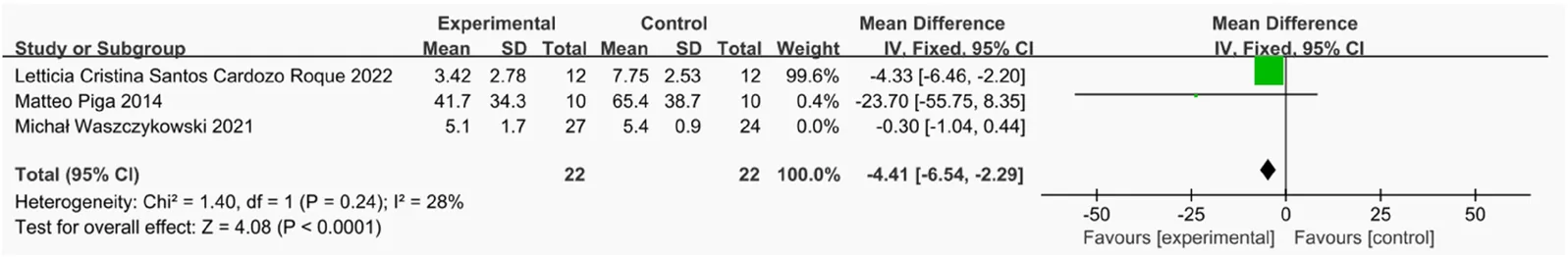
Forest map of rehabilitation exercise to reduce patient pain.

Among the three included studies (14, 17, 19), initial pooled analysis indicated substantial heterogeneity (*P* = 0.0008, *I*^2^ = 86%). We intended to conduct subgroup analysis or meta-regression to identify sources of heterogeneity according to the Cochrane Handbook, but only three trials reported pain data, which cannot meet the minimum number of studies required for reliable stratified or regression analyses. We therefore performed comprehensive leave-one-out sensitivity analysis to test the stability of pooled results. The study (17) was confirmed as the primary source of high heterogeneity. After excluding this small-sample trial, heterogeneity decreased to (*P* = 0.24, *I*^2^ = 28%), and a fixed-effects model was adopted for subsequent pooling. The results demonstrated that the VAS score in the rehabilitation group was significantly lower than that of the control group [MD = −4.41, 95% CI (−6.54, −2.29), *P* < 0.0001], as shown in Figure 1. It should be emphasized that excluding trial (17), which accounts for 33% of all pain-related data, only leaves two small-sample trials for fixed-effects pooling. This drastic loss of primary data severely weakens the statistical robustness of the pooled VAS pain estimate, and the pooled MD cannot be treated as reliable clinical evidence.

#### 3.3.3 Effect of rehabilitation intervention on the modified scleroderma hand mobility (HAMIS) test

**Figure 2:**
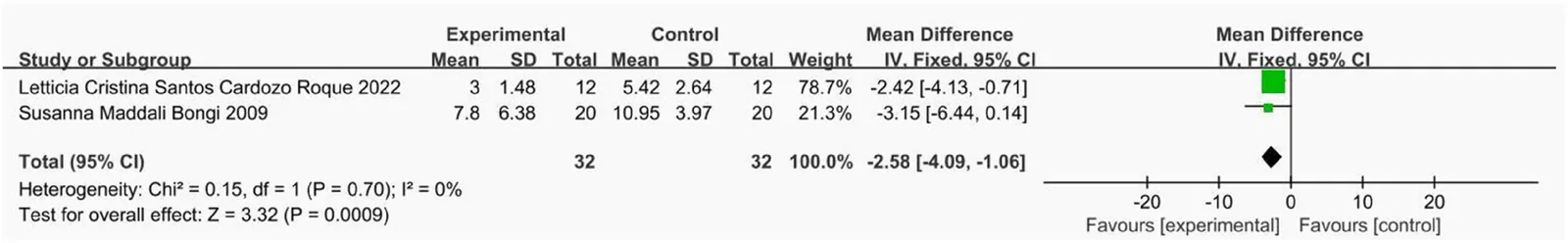
Forest map of hand range of motion improved by rehabilitation exercise.

Two studies (14, 19) evaluated the effect of rehabilitation exercise on the results of the HAMIS test. Heterogeneity testing indicated no significant heterogeneity among the studies (*P* = 0.70, *I*^2^ = 0%), and a fixed-effects model was employed for the meta-analysis. The results demonstrated that rehabilitation intervention significantly improved hand mobility [MD = −2.58, 95% CI (−4.09, −1.06), *P* = 0.0009], as shown in Figure 2.

#### 3.3.4 Effect of rehabilitation intervention on SHAQ score in patients with SSc

Two studies (19, 21) analyzed the SHAQ score. The heterogeneity test indicated no significant heterogeneity among the studies (*P* = 0.65, *I*^2^ = 0%), and a fixed-effects model was applied. The results demonstrated that the rehabilitation intervention did not yield a significant improvement in the SHAQ score [MD = −0.03, 95% CI (−0.25, 0.20), *P* = 0.81], as shown in Supplementary Figure 6. This result is reasonable because SHAQ is a multidimensional scale covering Raynaud's phenomenon, digital ulcers, gastrointestinal symptoms, pulmonary symptoms and overall disease severity, which is not specific to upper limb function alone. Local hand rehabilitation cannot alleviate systemic internal organ damage, so this neutral finding matched our prior expectation.

#### 3.3.5 Effect of rehabilitation intervention on DASH score in SSc patients

**Figure 3:**
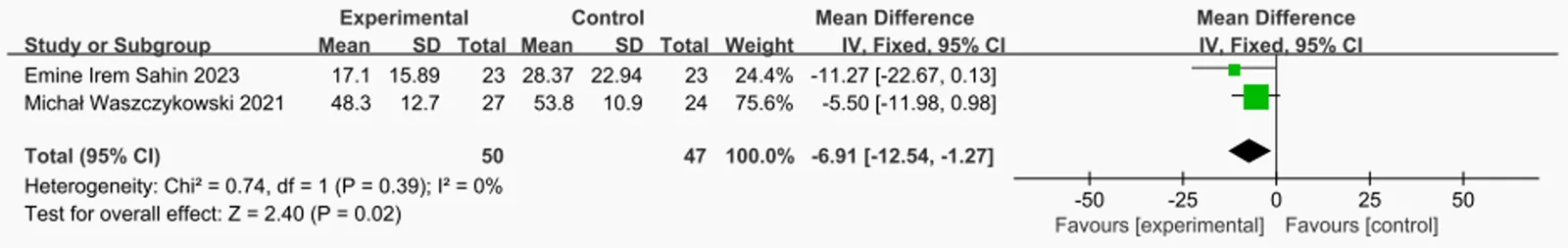
Forest plot of rehabilitation exercise to improve DASH score in patients.

Two studies (16, 17) analyzed the impact of rehabilitation exercise on the DASH score. Heterogeneity testing indicated no significant heterogeneity among the studies (*P* = 0.39, *I*^2^ = 0%), and a fixed-effects model was employed for the meta-analysis. The results demonstrated that the DASH score in the upper limb exercise group was significantly lower than that in the control group [MD = −6.91, 95% CI (−12.54, −1.27), *P* = 0.02], as shown in Figure 3.

### 3.4 Publication bias

A funnel plot of hand function data was generated for descriptive reference only Supplementary Figure 7. Per the Cochrane Handbook, at least 10 studies are required to reliably evaluate publication bias via funnel plots. Since only five trials reported hand function outcomes, the asymmetric funnel shape cannot yield credible evidence of publication bias, and no formal conclusions about unpublished literature are drawn from this graph.

## 4 Discussion

This meta-analysis aimed to investigate the effects of rehabilitation interventions on hand function, overall health, pain, and upper limb function in patients with systemic sclerosis (SSc), and to compare the effect differences between different delivery modes (in-person face-to-face vs. remote). The core findings indicate that rehabilitation exercise can significantly improve hand mobility and function, alleviate pain, and enhance upper limb function in SSc patients; however, its effect on improving the Scleroderma Health Assessment Questionnaire (SHAQ) score was non-significant. Furthermore, in-person face-to-face rehabilitation guidance showed a tentative favorable trend in improving hand dysfunction, but current evidence is underpowered and confounded, thus definitive superiority cannot be concluded. It should be noted that this meta-analysis only synthesized and compared macroscopic clinical functional scales, and was not designed to verify cellular or molecular pathological mechanisms. Brief tissue-level mechanistic inferences below draw on findings from published basic research, rather than core conclusions derived from our pooled clinical data.

The hands of patients with SSc are often significantly affected early in the disease course. Research (11) shows that patients rapidly experience progressive loss of joint mobility and muscle strength. The pathological evolution of hand skin lesions is characteristic: initially presenting as painless, non-pitting edema, progressing to skin thickening and hardening (presenting as a leathery change), and potentially leading to atrophy and thinning in the late stages (22). Common symptoms accompanying these changes include morning stiffness, joint pain, skin pigmentation abnormalities, telangiectasias, and subcutaneous calcinosis (23), which severely impair patients' activities of daily living and quality of life. Upper limb rehabilitation exercise holds multiple therapeutic values for SSc patients. Studies have confirmed that rehabilitation interventions may improve hand function reflected by the HAMIS scale, possibly through improving local microcirculation, relieving muscle tension, enhancing muscle strength, and increasing joint range of motion. Preclinical work further indicates that controlled physiological mechanical strain delivered via stretching and mobilisation may promote more ordered extracellular matrix organisation within fibrotic soft tissues, counteracting excessive tissue stiffness and contracture characteristic of SSc, though these tissue-level responses remain incompletely validated in human SSc hand trials. Appropriately dosed mechanical loading may modulate fibroblast mechanosignalling to limit pathological profibrotic matrix accumulation, yet immune-mediated fibrotic drivers may override such mechanical effects in active disease states. This functional improvement is consistent with a reduction in the Cochin Hand Function Scale (CHFS) score (24, 25), a finding further supported by the research of de Alegria et al.

Existing studies have hypothesized that the improvement in hand function from rehabilitation exercise may stem from its potential enhancement of local blood circulation. Previous studies have hypothesized that manual massage may elevate local skin temperature, which could facilitate mechanical perfusion and flow dynamics within blood and lymphatic vessels, accelerate the delivery of oxygen and nutrients to soft tissues, promote local metabolism, activate resting capillaries in skeletal muscle, and thereby alleviate muscle tension (17). Some studies indicate that combined hand exercises may maximize the range of motion of hand joints through stretching (14). Results from cohort studies also demonstrate that hand exercise programs are associated with better hand function, strength, and range of motion among SSc patients (26). Notably, however, this study found that the effect of rehabilitation interventions on improving the SHAQ score was limited. As SHAQ evaluates not only hand function but also Raynaud's phenomenon, digital ulcers, gastrointestinal symptoms, pulmonary symptoms, and overall disease severity (27), isolated upper limb rehabilitation cannot address multisystem lesions outside the extremities. Therefore, to improve patients' comprehensive health status and overall quality of life, multidisciplinary integrated interventions combining rheumatology, rehabilitation, gastroenterology and respiratory medicine are indispensable. Given the variations in SHAQ assessment methods among the included studies, future research requires more standardized assessment tools to enhance comparability of results.

Research (28) indicates that pain is one of the most common symptoms in SSc patients, with approximately 39% of patients reporting pain as a significant factor limiting their physical activity. The results of this study show that rehabilitation interventions (including whirlpool bath massage, active exercise, passive joint mobilization, etc.) are associated with significant pain relief. Previous clinical observations suggested that whirlpool baths may effectively promote hand blood circulation and relieve muscle tension; active exercise may enhance hand muscle strength and improve joint mobility; passive joint mobilization may help prevent joint contractures and maintain joint flexibility (8). Furthermore, rehabilitation exercise may potentially help alleviate pain and might reduce the occurrence of Raynaud's phenomenon and the risk of digital ulcers and amputations (29). From a pathophysiological perspective, rehabilitation exercise may possibly achieve pain relief by modulating inflammatory responses and decreasing the sensitivity of nerve endings. Such inferred mechanisms are consistent with findings from prior clinical investigations (30, 31). Regarding the improvement of upper limb function assessed by the DASH questionnaire, rehabilitation exercise showed a clear positive effect based on pooled clinical outcomes.

In-person face-to-face rehabilitation guidance showed a marginal trend in improving hand dysfunction in SSc patients, whereas the efficacy of remote rehabilitation was not demonstrated. It should be noted that this comparative result aligns with general clinical experience and does not provide a novel breakthrough finding, but it offers standardized evidence consolidation and methodological reflection for current clinical practice. Based on the delivery mode of the rehabilitation intervention, patients in the included studies were categorized into a remote rehabilitation exercise group and an in-person face-to-face rehabilitation guidance group. Meta-analysis results indicated a tentative advantage of in-person guidance; however, small sample size, heterogeneity in intervention protocol, supervision intensity and patient adherence render this comparison inconclusive. Notably, this observed between-group difference cannot be solely attributed to real-time supervision and corrective feedback, given co-intervention bias: many face-to-face protocols concurrently delivered manual soft-tissue mobilization that was absent from remote home-based programmes. While clinical plausibility exists for remote-mode limitations including uncorrected movement compensation and postural errors, existing trial data cannot isolate supervision-specific effects from additional hands-on physiotherapy components. Murphy et al. (32) suggested that remote rehabilitation may be hampered by the absence of real-time personalised adjustment mechanisms, yet this hypothesis remains unvalidated in SSc rehabilitation research. In the remote mode, due to the absence of on-site professional guidance, patients are prone to movement compensation and postural errors, directly affecting the biomechanical effectiveness of the rehabilitation training. Furthermore, the spatiotemporal limitations of remote communication make it difficult for therapists to promptly capture subtle functional changes in patients, thereby affecting the precise adjustment of the rehabilitation plan (33, 34).

Consistent with existing preclinical and rehabilitation research, structured supervised training delivers tangible benefits to soft tissue and peripheral circulation. Prior work focused on supervised motor training also confirms consistent structured practice improves neuromuscular coordination for fine hand movements (35). Targeted stretching eases persistent muscle tightness and facilitates joint lubrication through repeated active movement, which helps maintain cartilage health over time (36). Regular stretching regimens also boost peripheral angiogenesis and local blood flow to the hands, relieving tissue ischemia and limiting joint stiffness caused by subcutaneous calcium deposition (37). Guided aerobic training further improves microvascular responsiveness in the fingers, easing ischemic discomfort linked to Raynaud's phenomenon (38, 39).

At the practical level, therapists on-site can observe patients’ exercise movements in real time, promptly correct errors, and ensure the accuracy and effectiveness of the exercises. They can also flexibly adjust the rehabilitation plan based on the patient's specific performance and feedback during each session, providing more targeted guidance and psychological support, thereby enhancing patient compliance and motivation, and further contributing to the effective recovery of hand function (40, 41).

At the study design level, the included studies exhibited significant heterogeneity in sample size, participant characteristics, rehabilitation exercise modalities, and duration. The generalizability of conclusions from some small-sample studies is limited, and the diverse rehabilitation approaches make it difficult to comprehensively evaluate the overall efficacy. Regarding literature retrieval, searching only specific databases carries a risk of missing relevant studies, potentially leading to publication bias and affecting the objectivity of the results.

From an evidence strength perspective, the evidence supporting the improvement of hand function and pain relief through rehabilitation interventions in this study is relatively strong for local upper limb outcomes, as these conclusions are supported by consistent scale trends. However, the evidence strength regarding SHAQ score improvement and the efficacy of remote rehabilitation is relatively lower. The former is influenced by multidimensional attributes of SHAQ and inconsistent assessment methods, and the latter is limited by the small number of remote rehabilitation studies and their heterogeneity. Furthermore, the overall moderate methodological quality and absence of Grade A trials somewhat weaken the reliability of the evidence. Although the overall findings are largely consistent with existing clinical cognition, this study provides rigorous evidence sorting, methodological limitation appraisal, and actionable future research directions, which adds incremental value to clinical rehabilitation practice and subsequent trial design.

## 5 Conclusions

This meta-analysis found that rehabilitation exercise can significantly improve hand mobility and local hand function in scleroderma patients, and has positive effects on alleviating pain and enhancing upper limb function. No meaningful improvement was detected in the multi-system SHAQ metric, consistent with the limited action range of localized upper limb training. Isolated upper limb rehabilitation cannot address multisystem disease burden, highlighting the need for multidisciplinary intervention. Based on this, face-to-face rehabilitation guidance shows tentative clinical trends, which can only be regarded as a cautious reference for clinical practice. Specialists in physical and rehabilitation medicine (rehabilitation team coordinators) and licensed physical/occupational therapists, in conjunction with a multidisciplinary team including rheumatology, should develop personalized rehabilitation plans incorporating stretching and resistance training based on individual patient conditions, while strengthening patient education and exercise supervision. Community-based rehabilitation programs can be promoted to enhance patient participation convenience. Future research should focus on three aspects: first, prioritizing the implementation of in-person personalized rehabilitation, with multidisciplinary teams formulating plans and coordinating with rheumatology for primary disease management; second, exploring optimized pathways for remote rehabilitation through developing intelligent biofeedback motion correction systems to compensate for the lack of on-site supervision; third, conducting in-depth mechanistic studies and long-term follow-up, clarifying the mechanisms by which exercise improves microvascular function at the molecular level, and validating long-term efficacy through large-sample low-bias RCTs.

However, this study has certain limitations. At the study design level, significant heterogeneity existed among the included studies regarding sample size, participant characteristics, rehabilitation exercise modalities, and duration. A single large trial dominates many pooled results, and no high-quality Grade A trials are available, limiting the generalizability of conclusions from small-sample studies. Robust definitive clinical conclusions cannot be drawn based on the current evidence base. In terms of literature retrieval, searching only predefined electronic databases may lead to omitted relevant studies, which could introduce publication bias and compromise the objectivity of our findings. Only three trials reported pain outcomes, restricting us from performing subgroup analysis or meta-regression to explore the sources of high inter-study heterogeneity, which constitutes a key methodological limitation of this meta-analysis. Consistent with Cochrane methodological standards, the small pool of five trials assessing hand function means the funnel plot cannot deliver credible evaluation of publication bias. In addition, although we stratified analyses to separate passive manual therapy and active home exercise, the limited number of trials within each subgroup reduces the robustness of subgroup comparative results. This co-intervention bias means we cannot reliably disentangle the therapeutic contribution of manual manipulation from the independent effect of exercise supervision within these face-to-face trials. Future research should prioritize high-quality, low-risk-of-bias RCTs, standardize rehabilitation schemes and outcome measurement tools, implement long-term follow-up observation, and generate more robust evidence to guide clinical rehabilitation practice.

## Data Availability

The original contributions presented in the study are included in the article/Supplementary Material, further inquiries can be directed to the corresponding authors.
