# Supplementary material for: Systematic review and meta-analysis of the effectiveness of rehabilitation interventions on upper limb function in patients with systemic sclerosis
Source: Front Med (Lausanne). 2026 Sep 9;13:1899005. doi: 10.3389/fmed.2026.1899005 (PMC13597237; doi:10.3389/fmed.2026.1899005)
Supplement: Supplementary file 1 [file Datasheet1.docx]

Supplementary Material

# 1 Supplementary Figures and Tables

## 1.1 Supplementary Figures

**Supplementary Figure 1.**

Fig 1|Diagram of PubMed search strategy

**Supplementary Figure 2.**

Fig 2 |Flowchart of literature screening and inclusion

**Supplementary Figure 3.**

Fig 3|Proportion of projects with risk of bias in included studies

**Supplementary Figure 4.**

Fig 4|Risk of bias of included studies

**Supplementary Figure 5.**

Fig 5|Forest map of rehabilitation exercise to improve hand function in patients

Note: The pooled effect estimate of the in-person rehabilitation subgroup is subject to confounding bias from extra manual physical therapy and requires extremely cautious interpretation.

**Supplementary Figure 6.**

Fig 6|Rehabilitation exercise improved the forest map of SHAQ score in patients

## 1.2 Supplementary Table

**Basic characteristics of included literature**

|  |  |  | Sample Size (n) | |  | Age (years) | |  | Intervention | |  |  |
| --- | --- | --- | --- | --- | --- | --- | --- | --- | --- | --- | --- | --- |
| Study | Country | Intervention Duration (weeks) | T(F/M) | C(F/M) |  | T | C |  | T | C |  | Outcome Measures |
| Susanna Maddali Bongi et al.,2009^[14]^ | Italy | 9 | 20  (16/4) | 20  (14/6) |  | 56.4 ± 10.2 | 58.1 ±  13.4 |  | Physiotherapy  ① | Routine Intervention  ①③ |  | HAMI､  CHFS |
| Linda Kwakkenbos et al., 2022^[15]^ | Canada | 24 | 280  (250/30) | 186  (168/18) |  | 56.0 ± 12.7 | 55.4 ± 11.9 |  | Internet-Based Guided Training  ① | Routine Intervention  ③ |  | CHFS |
| Emine Irem Sahin et al., 2023^[16]^ | Turkey | 8 | 23(21/2) | 23(21/2) |  | 53.43 ± 11.95 | 57.60 ± 10.97 |  | Internet-Based Guided Training  ② | Routine Intervention  ② |  | DASH |
| Michał Waszczykowski et al., 2021^[17]^ | Poland | 4 | 27(25/2) | 24(22/2) |  | 54.5 ± 10.1 | 55.2 ± 9.2 |  | Physiotherapy  ② | Routine Intervention  ① |  | DASH､VAS､CHFS |
| Marie-Eve Carrier et al., 2022^[18]^ | Netherlands | 12 | 24(21/3) | 16(15/1) |  | 57.1 ± 13.5 | 58.7 ±  16.8 |  | Internet-Based Guided Trainin  ① | Routine Intervention  ③ |  | CHFS |
| Letticia Cristina Santos Cardozo Roque et al., 2022^[19]^ | Brazil | 12 | 12(12/0) | 12 (10/2) |  | 44.08±10.60 | 50.75 ± 11.09 |  | Physiotherapy  ③ | Routine Intervention ② |  | CHFS､VAS､HAMIS､SHAQ |
| Matteo Piga et al., 2014^[20]^ | Italy | 12 | 10 | 10 |  | 57.0±10.0 | 57.4 ± 11.7 |  | Internet-Based Guided Training  ③ | Routine Intervention  ① |  | VAS |
| Tugba Civi Karaaslan et al., 2023^[21]^ | Turkey | 8 | RTT: 14 (12/2)  AT: 14 (13/1) | 14(14/0) |  | RTT:41.86±10.76  AT:  47.14±10.76 | 43.50 ± 11.71 |  | Internet-Based Guided Training  ② | Routine Intervention ③ |  | DASH､SHAQ |

Notes: I. Assessment Tools: CHFS: Cochin Hand Function Scale; HAMIS:Hand Mobility in Scleroderma (modified); VAS: Visual Analog Scale (cm); SHAQ: Scleroderma Health Assessment Questionnaire; DASH: Disabilities of the Arm, Shoulder and Hand Questionnaire.

RTT: Real-Time Telerehabilitation Group; AT: Asynchronous Telerehabilitation Group.

II. Physiotherapy: ①.Connective tissue massage and McMennell joint manipulation ②.Upper limb whirlpool bath massage and active exercises for the hand and elbow joints ③. Joint mobilization techniques, along with active and resisted active exercises for both hands.

III. Internet-Based Guided Training: ①.Website tracking ②.Telephone check-ins ③.Device monitoring.

IV. Routine Intervention (primarily included): ①.Home-based daily exercises:Basic training such as finger flexion/extension and wrist joint movements ②.Health counseling and education:Provision of an SSc healthcare handbook and disease management advice (e.g., skin care, joint protection knowledge) ③.Basic medical support: Conventional pharmacological treatment (e.g., vasodilators, immunosuppressants) and outpatient follow-up.

Ⅴ. Some in-person rehabilitation trials delivered additional massage, joint mobilization or hydrotherapy exclusively to intervention groups, which introduces confounding co-intervention bias. Only telerehabilitation trials adopted identical home-based hand exercise protocols for both arms, which could isolate the independent effect of supervision mode.
